# A novel biomarker signature based on circulating transfer RNA (tRNA) can detect and differentiate HIV-2 infection from HIV-1

**DOI:** 10.1128/jcm.00378-26

**Published:** 2026-08-31

**Authors:** Santanu Biswas, David R. McGivern, Indira K. Hewlett, Krishnakumar Devadas

**Affiliations:** 1 Laboratory of Molecular Virology, Division of Emerging and Transfusion Transmitted Diseases, Center for Biologics Evaluation and Research, Food and Drug Administration 4137, Silver Spring, Maryland, USA; St Jude Children's Research Hospital, Memphis, Tennessee, USA

**Keywords:** plasma tRNA, circulatory biomarker, area under the ROC curve, logistic regression analysis, predictive model

## Abstract

HIV-1 and HIV-2 share transmission and replication characteristics, but require clinical differentiation. HIV-2 exhibits lower viral loads and slower disease progression, and most patients remain long-term non-progressors. Differentiation is crucial for selecting appropriate viral load monitoring tests and avoiding ineffective antiretroviral treatments, particularly non-nucleoside reverse transcriptase inhibitors, and certain protease inhibitors that do not work against HIV-2. Increased PrEP use may result in new infections testing negative for viral markers. This study aims to identify circulating tRNA expression profiles that could serve as non-viral biomarkers to detect and differentiate HIV-2 from HIV-1 infections. An initial pilot study analyzed 88 tRNAs in plasma samples from HIV-1- (*n* = 4) and HIV-2 (*n* = 4)-infected individuals, plus four healthy controls, using a tRNA-based PCR array for target identification. Eighteen selected tRNAs were further analyzed in a larger independent group of samples: HIV-1 (*n* = 20), HIV-2 (*n* = 20), and healthy controls (*n* = 20) for independent verification. A plasma tRNA diagnostic model was developed from these results and evaluated in 24 independent samples to detect and differentiate HIV-2 from HIV-1 infection. We identified a tRNA-based model (Model-II) containing six differentially expressed tRNAs (Leu-TAA, mt-Asp-GTC, mt-Ala-TGC, mt-Lys-TTT, mt-LeuT-AA, and Gly-CCC-1) that could detect HIV-2 infection and distinguish it from HIV-1 infection and healthy controls with 100% accuracy, sensitivity, and specificity. We also found that other blood-borne viruses such as HTLV, HBV, and HCV had no cross-reactivity with HIV-2, demonstrating that Model-II is specific for HIV-2 infection. This study establishes proof of concept for tRNA expression profiles as HIV diagnostic biomarkers. The tRNA-based Model-II represents a promising diagnostic tool for identifying HIV-2 infection and differentiating it from HIV-1.

**IMPORTANCE:**

HIV-2 infection requires accurate differentiation from HIV-1 to guide appropriate antiretroviral therapy, as HIV-2 exhibits intrinsic resistance to non-nucleoside reverse transcriptase inhibitors and certain protease inhibitors. Current diagnostic challenges include the absence of FDA-approved HIV-2 RNA quantification assays and potential false-negative results in LEVI (low-level viremia with evolving viral infection) syndrome patients on pre-exposure prophylaxis (PrEP), in whom viral markers may be undetectable. We developed a novel diagnostic approach using circulating transfer RNA (tRNA) expression profiles as host-derived, non-viral biomarkers. Our six-tRNA panel (Model-II) achieved 100% accuracy, sensitivity, and specificity in detecting HIV-2 infection while distinguishing it from HIV-1 and other blood-borne viral infections (HTLV, HBV, and HCV). This represents the first tRNA-based diagnostic model for HIV, offering a promising complementary tool for early detection and accurate viral differentiation, particularly in clinical scenarios where traditional viral markers are absent or unreliable.

## INTRODUCTION

Human immunodeficiency virus type 2 (HIV-2), predominantly found in West Africa, can also cause AIDS, with an estimated 2 million people infected worldwide (1). While HIV-2 is rare in the United States, representing <0.1% of new HIV diagnoses (2–4), it accounts for substantial infection burdens in specific regions: 1%–5% prevalence in West African countries (Guinea-Bissau, Senegal, Gambia), 2% of HIV infections in France, and 5% in Portugal due to historical and ongoing migration patterns (2–4). Globally, HIV-2 represents a significant public health challenge, particularly in resource-limited settings where diagnostic infrastructure is limited (5).

HIV-1 and HIV-2 are closely related viruses that share many similar traits such as modes of transmission, viral replication, and pathogenesis. However, the differences between HIV-1 and HIV-2 patients are crucial because HIV-2 patients have a higher CD4+ T cell count at the time of AIDS onset and generally survive longer after AIDS onset (6). A recent study found that most people infected with HIV-2 remain long-term non-progressors, and the mean time for the progression to AIDS was 14.3 years for HIV-2 compared to 6.2 years for HIV-1. Similarly, the progression time to death was longer for HIV-2 compared to HIV-1 (15.2 years vs 8.2 years) (7). It is important to find the best diagnostic test to differentiate between HIV-1 and HIV-2 infection to prevent the use of inappropriate antiretroviral medications in HIV-2-infected individuals, including non-nucleoside reverse transcriptase inhibitors and some protease inhibitors that are ineffective against HIV-2 (1, 8, 9).

Current FDA-approved diagnostic tests for detecting HIV-2 infection use enzyme-linked immunosorbent and chemiluminescent immunoassay methods. While these immunoassays detect both HIV-1 and HIV-2, they do not differentiate between the two types of HIV infection. An FDA-approved rapid antibody test that detects HIV-2 infection uses an immunoconcentration method, incorporates peptide sequences representing HIV-1 and HIV-2 envelope proteins, and allows differentiation between HIV-1 and HIV-2 infections (10). An initial reactive result for serologic HIV-2 antibody test should be confirmed with a supplemental HIV-2 antibody test, such as an immunoblot. Recently, a few FDA-approved supplemental differentiation tests have become available for *in vitro* diagnostic use in the United States (11). However, some studies have identified cases where challenges remain in distinguishing the two viruses (12). To resolve ambiguous serologic results, there is one FDA cleared HIV-1/HIV-2 nucleic acid-based (NAT) qualitative test that detects and differentiates HIV-1 and HIV-2 RNA. However, there remain no FDA-approved assays for quantification of HIV-2 RNA. Certain HIV-1 RNA assays can occasionally identify HIV-2 RNA (13). In one study, only approximately 50% of HIV-2-infected patients had detectable HIV-2 RNA by reverse transcriptase polymerase chain reaction (PCR) testing, and among those patients with detectable RNA, the median viral load was 1,000 copies/mL (14). In the absence of a commercially available nucleic acid-based quantitative test, HIV-2 RNA levels cannot be monitored in patients who are on treatment. The development of an HIV-2 test is needed to improve the care of HIV-2-infected patients.

Individuals with acute HIV-1/HIV-2 infection are often unaware of their infection and may engage in high-risk behaviors facilitating HIV transmission (15). Therefore, early diagnosis is a key strategy for preventing HIV transmission. Individuals on highly suppressive antiretroviral therapy (ART) may have undetectable viral loads, making differentiation between HIV-1 and HIV-2 challenging when viral sequencing is not feasible due to low viremia. Additionally, the recently described LEVI syndrome, in which individuals initiating PrEP around the time of HIV exposure develop inconsistent serologic and nucleic acid test results, further complicates diagnosis (16, 17). Host-derived biomarkers, such as transfer RNA (tRNA) expression signatures, could provide complementary diagnostic information independent of viral replication levels, enabling HIV-1/HIV-2 differentiation even when viral markers are suppressed or fluctuating (18).

The landscape of HIV prevention has been transformed by the recent FDA approval of lenacapavir, a first-in-class, long-acting capsid inhibitor administered subcutaneously every six months for HIV-1 pre-exposure prophylaxis (PrEP). Clinical trials demonstrated >99% efficacy in preventing HIV-1 acquisition (19, 20). However, lenacapavir specifically targets the HIV-1 capsid protein and is less potent against HIV-2 (21). In West Africa, where HIV-2 prevalence ranges from 1% to 5% in some populations, and lenacapavir PrEP programs are being implemented, the need for robust HIV-2 diagnostic capabilities is urgent (21, 22). This unmet need motivates the development of novel, host-based biomarkers that can detect and differentiate HIV-2 infection independent of viral load or antibody status, complementing existing diagnostic algorithms.

Host tRNA is a small molecule ribonucleic acid that carries and transports amino acids and consists of a short chain of 70–90 nucleotides in length folded into a cloverleaf shape (23). Transfer RNAs (tRNAs) have emerged as a promising new class of biomarkers, exhibiting high relative abundance across a wide range of biofluids—including plasma, serum, saliva, cerebrospinal fluid, and urine—often exceeding the abundance of the more widely studied microRNAs (24–26). Furthermore, tRNAs are enriched in exosomes, where they constitute a major fraction of the small non-coding RNA content, and their stability in biofluids such as plasma is conferred by extensive post-transcriptional chemical modifications and tight tertiary folding, making them particularly attractive candidates for liquid biopsy applications (27). In recent years, studies have confirmed that in addition to amino acid transport, tRNA can also participate in the regulation of cell biological functions by affecting cell proliferation (28), differentiation (28), apoptosis (29), and metabolism (30). Alterations of tRNA repertoire affect cell fate during development. Many diseases show disruptions in the levels and distributions of tRNAs, such as type 2 diabetes mellitus (31), Huntington disease (32), and HIV infection (33). Viruses are wholly dependent on the host translation machinery to synthesize their proteins. After profiling the tRNA repertoire, earlier studies found that influenza A, hepatitis E, and vaccinia viruses can manipulate tRNA populations to favor translation of their own genes (34, 35). The codon usage of HIV-1 early genes is similar to that of highly expressed human genes, whereas the codon usage of the late genes is better adapted to the altered tRNA pool induced late in viral infection (33). However, to the best of our knowledge, the diagnostic utility of tRNA in HIV-1/HIV-2-infected patients has not been reported. To date, the relationship between tRNA expression in HIV-1 and HIV-2 infection is poorly understood. In our study, we first investigate the expression profile of tRNA in HIV-1- and HIV-2-infected patients and evaluate the diagnostic utility of tRNA. More importantly, we demonstrate the diagnostic relevance of specific tRNA in this study to establish a tRNA-based diagnostic model to differentiate HIV-2 from HIV-1 infection.

## MATERIALS AND METHODS

### Patient information

Seroconversion panels were purchased from SeraCare Life Sciences, Gaithersburg, MD, USA (eight panels containing eight HIV-1- positive plasma samples), and ZeptoMetrix Corp., Buffalo, NY, USA (two seroconversion panels containing two HIV-1- positive plasma samples). Four HIV-1- positive post-seroconversion p31-positive plasma samples and two AccuSet HIV-1 performance panels containing 18 HIV-1- positive plasma samples were purchased from SeraCare Life Sciences. Thirty-two HIV-2- positive plasma samples were purchased from Boca Biolistics, LLC, Pompano Beach, FL, USA. All HIV-1- positive samples were reactive by HIV-1 antibody test, with a reactive or indeterminate Western blot or positive Western blot reactivity. All HIV-2- positive samples were from individuals who were diagnosed as HIV-2- positive according to the serological algorithms. All plasma specimens from HIV-1/HIV-2-infected individuals were characterized by the vendors with commercially available FDA-approved/licensed assays and in-house research use only (RUO) assays. Among the 32 HIV-2- positive samples, nine samples exhibited a viral load of less than 100 copies per mL, as determined by the laboratory-developed PCR test provided by the vendor.

HCV- and HBV-positive plasma samples were purchased from American Red Cross, Gaithersburg, MD, USA. AccuSet HTLV-I/II performance panel was purchased from SeraCare Life Sciences.

Human whole blood (WB) samples were collected by venipuncture into EDTA-K2 tubes (BD Vacutainer, Franklin Lakes, NJ, USA) from 36 healthy volunteer donors at the National Institutes of Health (NIH) Division of Transfusion Medicine. All samples tested seronegative for HIV, hepatitis B, and hepatitis C virus. This protocol was approved by the NIH ethics committee (study number: 99-CC-0168). To prepare plasma, WB samples were centrifuged three times at 4°C (1,500 rpm for 30 min, 3,000 rpm for 5 min, and 4,500 rpm for 5 min) (36). Plasma samples were stored at −80°C until use.

### Study design

**Fig 1:**
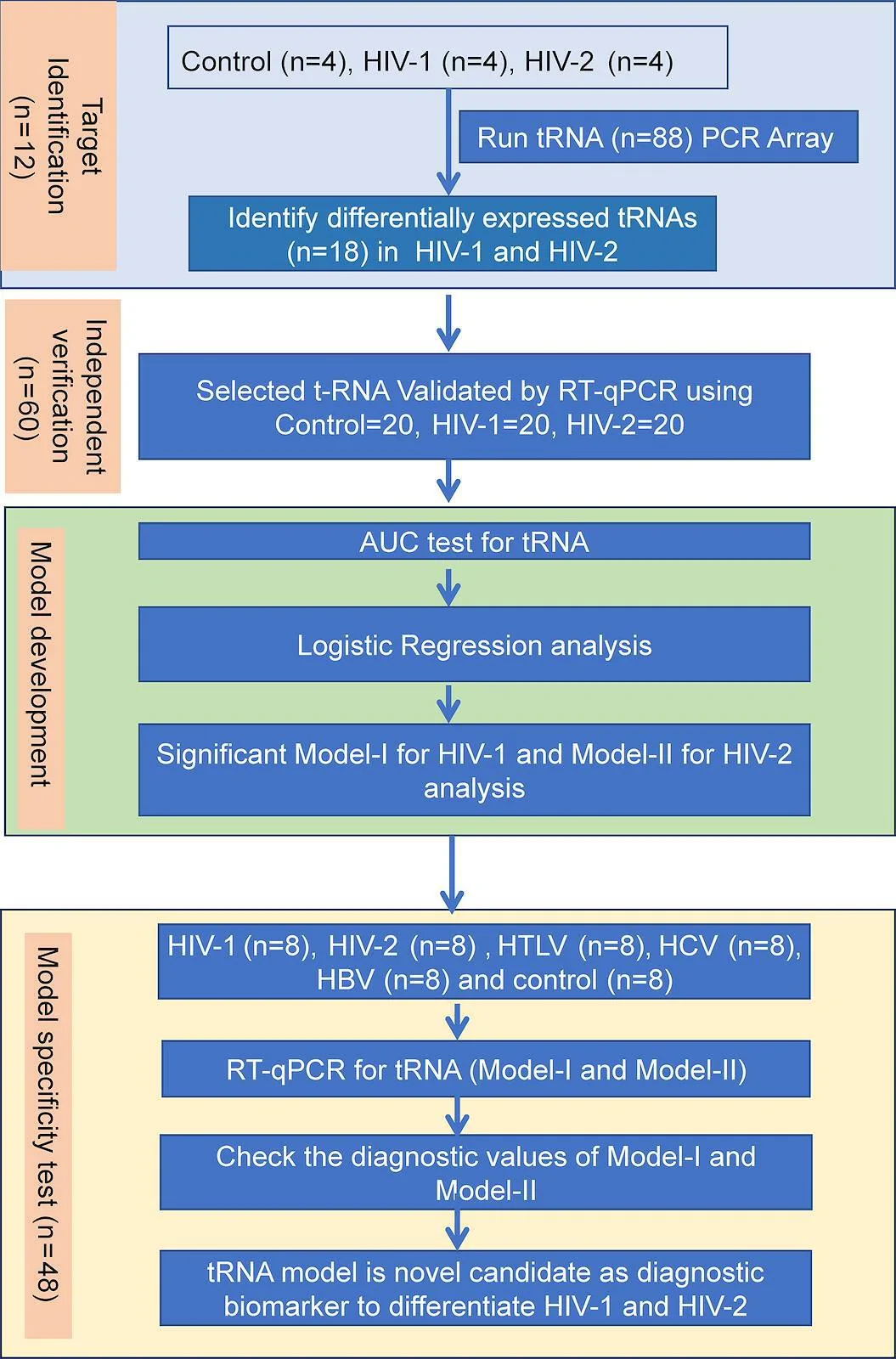
Study design. The transfer RNA (tRNA) profiles of 120 plasma samples from 24 HIV-1- and 24 HIV-2-infected individuals and 24 healthy controls were used to generate outcomes in two different stages. The candidate tRNAs identified in 12 plasma samples using tRNA PCR arrays were further verified in 60 plasma samples using quantitative RT-PCR. The logistic regression and ROC curve analyses were performed in the model development stage. The models were evaluated in the model specificity test phase using 48 blinded plasma samples.

The experimental plan was divided into four stages (Fig. 1): target identification, independent verification, model development, and model testing. During the target identification stage, samples were screened using the nrStar Human tRNA PCR Array v 1.0 (AS-NR-001H-1), and promising differentially expressed tRNAs were identified and chosen for further study. In the independent verification stage, the selected candidate tRNAs were further evaluated. In the model development stage, the verified tRNAs were combined to form a diagnostic model. During the independent verification stage, the predictive performance of the tRNA panels to serve as possible biomarkers was also evaluated. The clinical diagnostic performance of the tRNA diagnostic models was examined in the model specificity test stage with samples whose clinical diagnostic information was blinded to us. Diagnostic information, sensitivity, and specificity of the logistic models in distinguishing HIV-1 infection and HIV-2 infection from healthy control participants were estimated after unblinding the test findings.

### Total RNA extraction from plasma

Total RNA was isolated from plasma as previously described (37) using the miRNeasy Serum/Plasma Kit (Cat no. 217184 Qiagen, USA). Briefly, a 200 μL of plasma was lysed with 1 mL of QIAzol and homogenized by vigorous mixing on a vortex mixer (15 s) and then incubated at room temperature (RT) for 5 min. Finally, RNA was eluted by adding 16 µL of DNase/RNase-free water to the column, followed by centrifugation at 13,000 × *g* for 1 min at RT. Eluted total RNA samples were stored at −80°C until use.

### Demethylation and first-strand cDNA synthesis

From the eluted RNA, 14 μL RNA was used for demethylation and subsequent cDNA synthesis using the rtStar tRNA-optimized First-Strand cDNA Synthesis Kit (Arraystar, Rockville, MD, USA). After termination of the demethylation reaction, RNA was precipitated with 75% ethanol and dissolved in 12 μL of RNase-free water. To synthesize cDNA, 10 μL of demethylated RNA was used according to the manufacturer’s instructions.

### Quantitative polymerase chain reaction

cDNA was diluted (1:40) in nuclease-free water, followed by qPCR using the nrStar Human tRNA Repertoire PCR Array (Arraystar Rockville, MD, USA) and the Applied Biosystems ViiA7 Real-Time PCR System (Thermo-Fisher Scientific, USA), according to the manufacturer’s instructions. The primers (Arraystar, Inc.) were specifically designed to amplify full-length mature tRNAs (70–90 nucleotides). The nrStar Human tRNA PCR Array profiles 88 tRNAs, including 66 nuclear-encoded tRNAs and 22 mitochondrial-encoded tRNAs based on their genomic origin. Primers were designed to target tRNA sequences that correspond to nuclear or mitochondrial genes. Throughout this manuscript, we use the terms “nuclear encoded” and “mitochondrial encoded” to refer to the genomic origin of the tRNA gene sequences detected by our primers, not their cellular localization. For further validation, the primers of 17 candidate tRNAs and 18S rRNA as a housekeeping gene were used (Arraystar, Inc. Rockville, MD, USA). The expression levels of tRNAs were determined by the ΔCt method using 18S rRNA as an internal control.

### tRNA-based diagnostic model

The association between tRNA expression and HIV-1/HIV-2 was analyzed. Transfer RNA-based diagnostic models were designed to predict the HIV-1 or HIV-2 infection. Those tRNAs that were differentially expressed between HIV-1/HIV-2 and normal controls were selected as candidates in the regression analysis. A univariate analysis was performed for each candidate tRNA, and differentially expressed tRNAs (*P* < 0.05) were included in a multivariate analysis. A tRNA-based logit score was calculated with these tRNAs, weighted by their estimated regression coefficients. The receiver operating characteristic (ROC) curve was plotted to demonstrate the sensitivity and specificity of the tRNA-based diagnostic model. Area under the curve was calculated from the ROC curves. Finally, the clinical diagnostic performance of the tRNA models established in the verification stage was further tested in the model specificity test stage. In this stage, the investigators were blinded to the clinical diagnostic test results of the 48 plasma samples. After unblinding the clinical diagnostic information, the sensitivity and specificity of the logistic models in discriminating HIV-1 infection and HIV-2 infection from healthy subjects were calculated. The samples used in the verification stage and model specificity test stage were independent of the target identification stage and were also independent of each other.

### Statistical analysis

Power analysis calculations were performed using G*Power 3.1 (38). In the pilot study/target identification stage with *n* = 4 per group, we achieved >75% power to detect 4-fold changes at *α* = 0.05. The independent verification stage, with *n* = 20 per group, provided >90% power at *α* = 0.05. The blinded validation phase (*n* = 8 per group) was designed to assess real-world diagnostic performance, achieving 100% sensitivity and specificity for Model-II (95% CI: 63.1%–100% by exact binomial method).

Significance analysis of the data obtained from the PCR arrays was used to identify the differentially expressed tRNAs between HIV-1/HIV-2-infected individuals and healthy normal controls. Student’s *t*-test was used to compare differences in plasma tRNA expression levels between the HIV-1-/HIV-2-infected group and the healthy normal control group. Receiver operating characteristic (ROC) curves and the area under the ROC curve (AUC) were used to assess the performance of the selected tRNAs to serve as diagnostic tools/biomarkers for detecting HIV-1 or HIV-2 infection. Logistic regression was used to develop a linear combination of tRNAs. The AUC was used as an accuracy index for evaluating the diagnostic performance of the selected models. The optimal cutoff point for the diagnostic tRNA models was determined by the Youden index. The statistical results were expressed by 95% confidence intervals (CIs). All tests were two-tailed. Significance was set at *P* ≤ 0.05. We used SPSS version 20.0 Software (SPSS Inc., Chicago, IL, USA) and GraphPad Prism 10.0 (GraphPad Software, Inc., California, USA).

## RESULTS

### Differences in tRNA expression profiles between HIV-1 and HIV-2 infection

To identify circulating tRNA that could serve as biomarkers to detect and differentiate between HIV-1 and HIV-2 infections, we analyzed a total of 96 plasma samples from HIV-1-infected patients, HIV-2-infected patients, and normal controls. The overall approach is detailed in Fig. 1.

**Fig 2:**
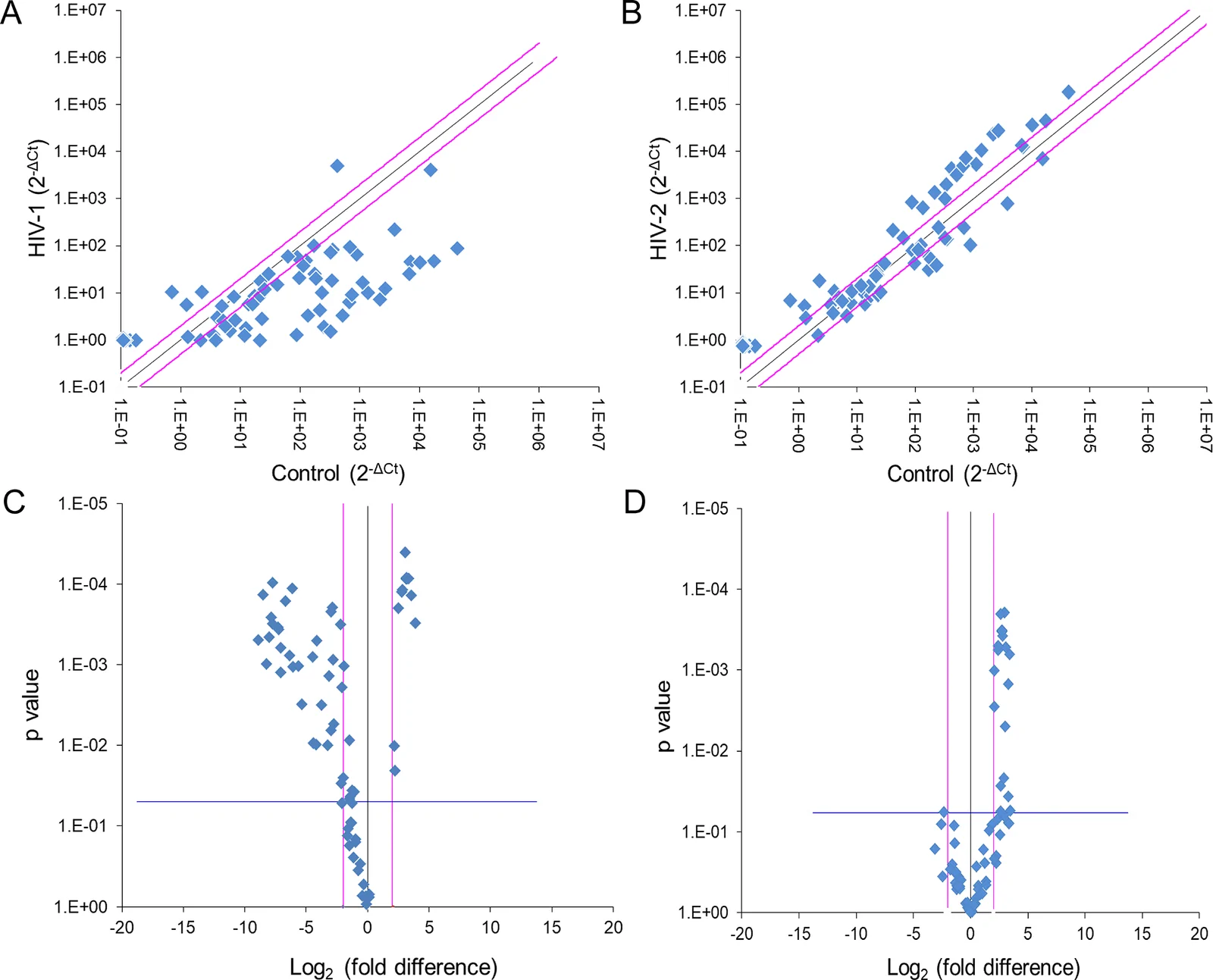
Visualization of differentially expressed tRNAs in plasma from persons infected with HIV-1 or HIV-2 compared to plasma from uninfected healthy donors. Scatter plots show differentially expressed tRNAs in (**A**) HIV-1 versus uninfected normal control and (**B**) HIV-2 versus uninfected normal control. Each blue dot represents an individual tRNA. The black line indicates fold change (2 ^ (−ΔCt)) of 1 (no difference). The pink lines indicate the 2-fold change threshold. The axes show the mean normalized tRNA signal values for each comparator group. The tRNAs lying above and below these pink lines displayed greater than a 2-fold upregulation or downregulation. Volcano plots show differentially expressed tRNAs in (**C**) HIV-1 versus uninfected normal control and (**D**) HIV-2 versus uninfected normal control. The *x*-axis represents fold-change values (log2 scaled), while the *y*-axis represents *P*-values (−log10 scaled). The pink vertical lines correspond to 2-fold upregulation and downregulation, respectively, while the blue horizontal line corresponds to a *P*-value of 0.05. On this basis, the blue dots on the right-hand side and left-hand side quadrants represent the statistically significant upregulated and downregulated tRNAs, respectively.

**Fig 3:**
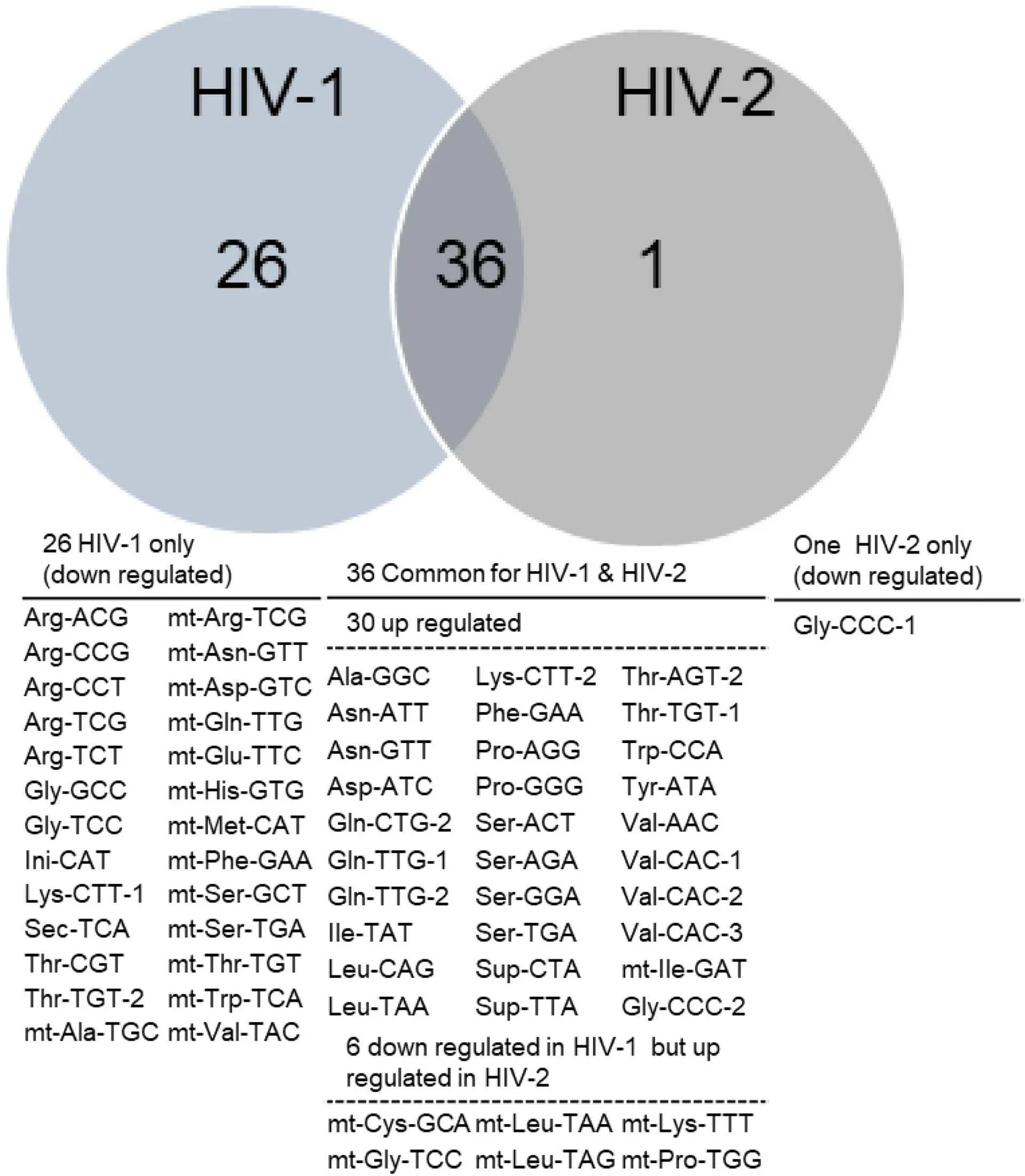
Venn diagram of differentially expressed tRNAs generated from PCR array data, illustrating the overlap of upregulated or downregulated tRNAs between HIV-1-infected individuals vs controls and HIV-2-infected individuals vs controls.

In the first stage of this analysis, plasma RNA from four normal controls, four HIV-1- positive, and four HIV-2- positive plasma samples was amplified, and the expression levels of 88 tRNAs (66 nuclear-encoded tRNAs and 22 mitochondrial-encoded tRNAs) were profiled using the nrStar Human tRNA PCR array V 1.0 (AS-NR-001H-1, Arraystar Inc., Rockville, MD, USA). We compared the levels of tRNAs between HIV-1 vs controls and HIV-2 vs controls (Fig. 2 and Table S1). The scatter plots in Fig. 2A and B are used to visualize differences in the normalized signal values of the tRNAs between groups. Figure 2C and D show the volcano plots for the differentially expressed tRNAs in uninfected control vs HIV-1 infection and uninfected control vs HIV-2 infection, respectively. Data from PCR array analysis indicated that 62 tRNAs (30 upregulated and 32 downregulated) were differentially regulated in the HIV-1-infected population, and 37 tRNAs (36 upregulated and 1 downregulated) were differentially regulated in the HIV-2-infected population. Differentially regulated tRNAs were defined as those showing a greater than or less than log_2_ 2-fold change in expression with a *P*-value of ≤0.05. The complete list of differentially regulated tRNAs from the HIV-1/HIV-2-infected groups compared to the uninfected control group is provided in Table S1. Results from the PCR array analysis showed that 26 and 1 downregulated tRNAs were unique to the HIV-1 and HIV-2 infected individuals, respectively (Fig. 3). Thirty tRNAs (Fig. 3) were upregulated in both the HIV-1- and HIV-2-infected groups. Surprisingly, six tRNAs (mt-Cys-GCA, mt-Gly-TCC, mt-Leu-TAA, mt-Leu-TAG, mt-Lys-TTT, and mt-Pro-TGG) were significantly downregulated in the HIV-1-infected population but were significantly upregulated in the HIV-2-infected population (Fig. 3), suggesting that they play different roles in the regulatory mechanisms of infectivity and replication in HIV-1 and HIV-2 infection.

HIV-1 and HIV-2 infections show distinct patterns of tRNA expression compared to control individuals and between each other. HIV-1 infection is associated with relatively lower expression of specific isoaccepting tRNAs, including arginine tRNAs (Arg-ACG, Arg-CCG, Arg-CCT, Arg-TCG, Arg-TCT), glycine tRNAs (Gly-GCC, Gly-TCC), threonine tRNAs (Thr-CGT, Thr-TGT-2), mitochondrial-encoded tRNAs for glutamine (mt-Gln-TTG, mt-Glu-TTC), leucine (mt-Leu-TAA, mt-Leu-TAG), and serine (mt-Ser-GCT, mt-Ser-TGA) (Table S1.1). In contrast, HIV-2 infection is associated with relatively higher expression of different isoaccepting tRNAs, including asparagine tRNAs (Asn-ATT, Asn-GTT), glutamine tRNAs (Gln-CTG-2, Gln-TTG-1, Gln-TTG-2), leucine tRNAs (Leu-CAG, Leu-TAA), serine tRNAs (Ser-ACT, Ser-AGA, Ser-GGA, Ser-TGA), valine tRNAs (Val-AAC, Val-CAC-1, Val-CAC-2), and mitochondrial-encoded leucine tRNAs (mt-Leu-TAA, mt-LeuTAG) (Table S1.2).

### Expression profile of tRNAs verified by qRT-PCR

**Fig 4:**
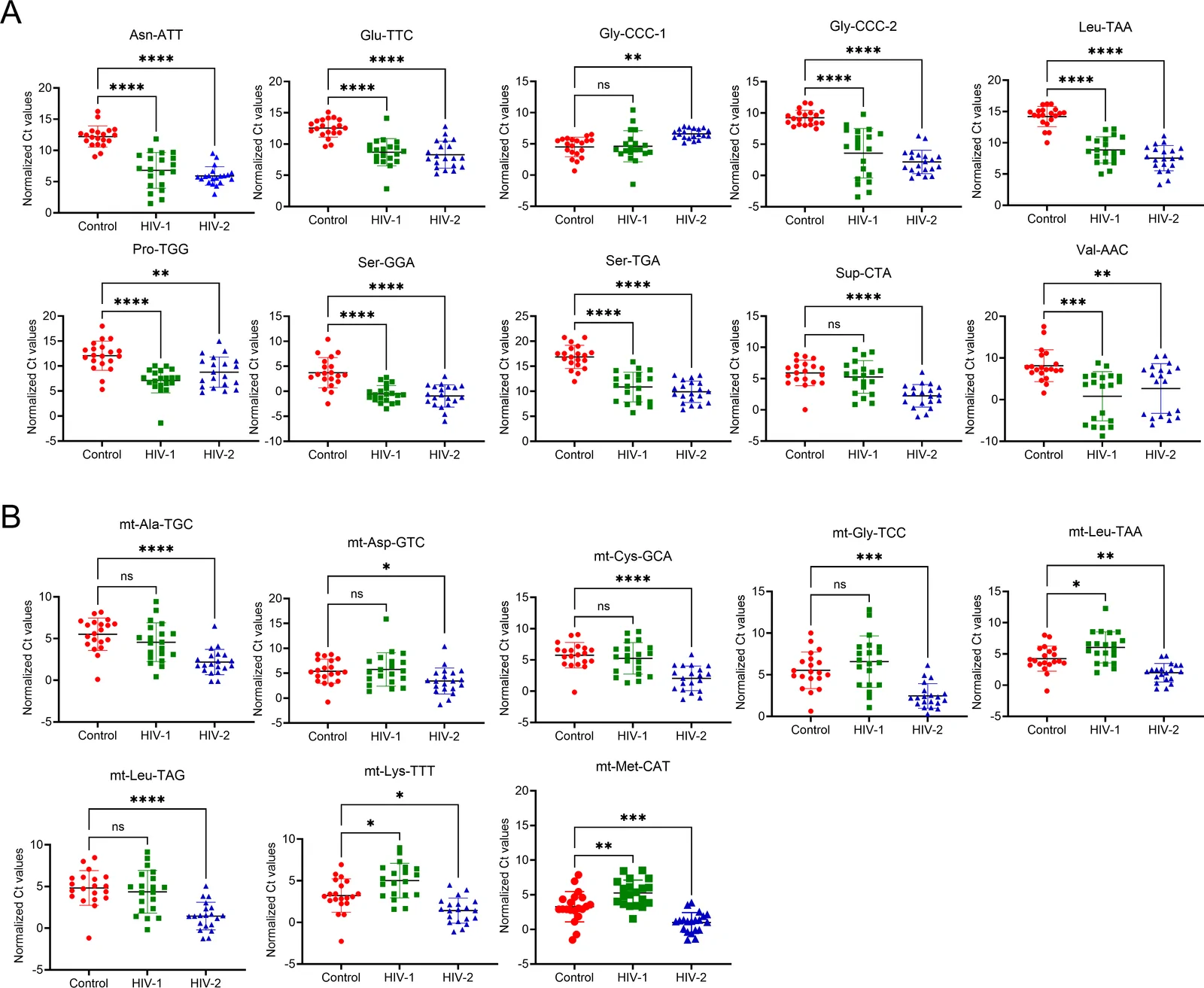
Verification of the circulating transfer RNAs (tRNAs) expression profiles in the independent sample set. Expression levels of (**A**) nuclear-encoded and (**B**) mitochondrial-encoded tRNAs in the plasma of HIV-1- and HIV-2-infected patients. The levels of tRNAs in plasma samples were from individuals infected with HIV-1 (*n* = 20) and HIV-2 (*n* = 20), and 20 uninfected control plasma samples were examined using real-time RT-PCR and normalized to 18S rRNA as the internal control. Comparison of the tRNA expression profiles in plasma samples from HIV-1- and HIV-2-infected individuals with those from uninfected controls indicated that the expression of nine nuclear-encoded tRNAs (**A**) was significantly increased and three mitochondrial-encoded tRNAs (**B**) were significantly decreased in the plasma from HIV-1-infected individuals. On the other hand, seven mitochondrial-encoded tRNAs (**B**) were significantly increased, and 10 nuclear-encoded tRNAs (**A**) were significantly changed compared to the uninfected controls. (*P ≤ 0.05; **P ≤ 0.01, ***P ≤ 0.0001, and ns, non-significant, as calculated using Student’s *t*-test).

To identify tRNA profiles with the potential to distinguish HIV-1 and HIV-2, further qPCR analyses were conducted with a focus on the 18 tRNAs (Table S2) that were differentially expressed in HIV-1 - and HIV-2-infected patients. These tRNAs were chosen based on the fold change between the groups being studied and their low Ct values, which suggested that they could be abundant and readily measurable in plasma. The selected tRNAs were further verified in a larger independent sample (40 plasma samples from HIV-1/HIV-2-infected individuals and 20 uninfected controls) using quantitative RT-PCR and 18S rRNA as internal controls for normalization. Comparison of the tRNA expression profiles in plasma samples from HIV-1-infected individuals with those from uninfected controls indicated that the expression of eight nuclear-encoded tRNAs was significantly increased and three mitochondrial-encoded tRNAs were significantly decreased in the plasma from HIV-1-infected individuals (Fig. 4; Table S3). In HIV-2 infection, 18 tRNAs were significantly upregulated (10 nuclear-encoded tRNAs and eight mitochondrial-encoded tRNAs) when compared to uninfected controls (Fig. 4; Table S3).

### Diagnostic value of circulating tRNAs

**Fig 5:**
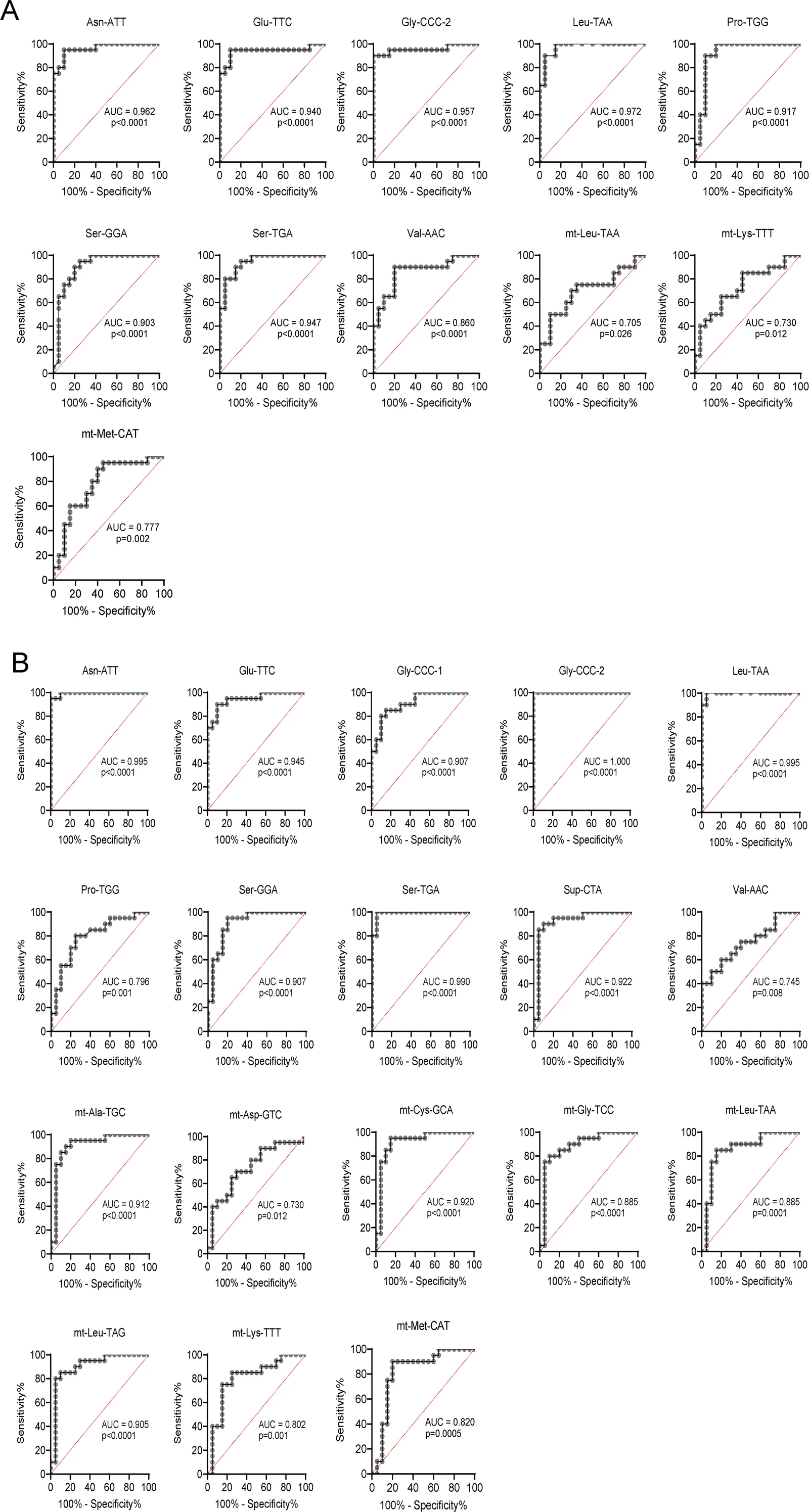
Receiver operating characteristic (ROC) curve analysis of sensitivity and specificity of plasma tRNAs in distinguishing (**A**) HIV-1 infection from uninfected control and (**B**) HIV-2 infection from uninfected control. The levels of plasma tRNAs in HIV-1 (*n* = 20), HIV-2 (*n* = 20), and uninfected control patient (*n* = 20) groups were subjected to ROC analyses.

The diagnostic accuracy of these tRNAs was evaluated using ROC analysis for detecting HIV-1 infection (Fig. 5 and Table 1). Our results showed that Asn-ATT, Glu-TTC, Gly-CCC-2, Leu-TAA, Pro-TGG, Ser-GGA, Ser-TGA, Val-AAC, mt-Leu-TAA, mt-Lys-TTT, and mt-Met-CAT were significantly modulated and were able to discriminate individuals with HIV-1 infection from normal uninfected control population. These tRNAs had AUC values of 0.962, 0.940, 0.957, 0.972, 0.917, 0.903, 0.947, 0.860, 0.705, 0.730, and 0.777, respectively (Fig. 5A and Table 1). Our results also showed that Asn-ATT, Glu-TTC, Gly-CCC1, Gly-CCC2, Leu-TAA, Pro-TGG, Ser-GGA, Ser-TGA, Sup-CTA, Val-AAC, mt-Asp-GTC, mt-Ala-TGC, mt-Cys-GCA, mt-Gly-TCC, mt-Leu-TAA, mt-Leu-TAG, mt-Lys-TTT, and mt-MetCAT could significantly distinguish HIV-2 infection from the uninfected control population, and the AUC values for these tRNAs were 0.995, 0.945, 0.907, 1.000, 0.995, 0.796, 0.907, 0.990, 0.922, 0.745, 0.912, 0.730 0.920, 0.885, 0.855, 0.905, 0.802, and 0.820, respectively (Fig. 5B and Table 1).

**TABLE 1:** Diagnostic performance of individual tRNAs for detection of HIV-1 and HIV-2 infection

Name of the tRNA	AUC (95% CI)	*P*-value^*a*^
HIV-1 vs control group		
Asn-ATT	0.962 (0.910–1.000)	<0.0001
**Glu-TTC**	**0.940 (0.850–1.000**)	**<0.0001**
Gly-CCC-1	0.532 (00346–0.718)	0.725
Gly-CCC-2	0.957 (0.887–1.000)	<0.0001
Leu-TAA	0.972 (0.930–1.000)	<0.0001
**Pro-TGG**	**0.917 (0.817–1.000**)	**<0.0001**
Ser-GGA	0.903 (0.801–1.000)	<0.0001
Ser-TGA	0.947 (0.885–1.000)	<0.0001
Sup-CTA	0.567 (0.385–0.749)	0.465
**Val-AAC**	**0.860 (0.741–0.978**)	**<0.0001**
mt-Ala-TGC	0.647 (0.471–0.823)	0.110
mt-Asp-GTC	0.505 (0.322–0.688)	0.956
mt-Cys-GCA	0.557 (0.373–0.741)	0.533
mt-Gly-TCC	0.607 (0.427–0.787)	0.244
**mt-Leu-TAA**	**0.705 (0.539–0.870**)	**0.026**
mt-Leu-TAG	0.570 (0.387–0.752)	0.448
mt-Lys-TTT	0.730 (0.572–0.887)	0.012
mt-Met-CAT	0.777 (0.629–0.925)	0.002
HIV-2 vs control group		
Asn-ATT	0.995 (0.981–1.000)	<0.0001
Glu-TTC	0.945 (0.877–1.000)	<0.0001
**Gly-CCC-1**	**0.907 (0.819–0.995**)	**<0.0001**
Gly-CCC-2	1.000 (1.000–1.000)	<0.0001
**Leu-TAA**	**0.995 (0.981–1.000**)	**<0.0001**
Pro-TGG	0.796 (0.655–0.937)	0.001
Ser-GGA	0.907 (0.812–1.000)	<0.0001
Ser-TGA	0.990 (0.967–1.000)	<0.0001
Sup-CTA	0.922 (0.825–1.000)	<0.0001
Val-AAC	0.745 (0.592–0.897)	0.008
**mt-Ala-TGC**	**0.912 (0.811–1.000**)	**<0.0001**
**mt-Asp-GTC**	**0.730 (0.571–0.889**)	**0.012**
mt-Cys-GCA	0.920 (0.824–1.000)	<0.0001
mt-Gly-TCC	0.885 (0.772–0.997)	<0.0001
**mt-Leu-TAA**	**0.885 (0.772–0.997**)	**0.0001**
mt-Leu-TAG	0.905 (0.801–1.000)	<0.0001
**mt-Lys-TTT**	**0.802 (0.658–0.946**)	**0.001**
mt-Met-CAT	0.820 (0.675–0.964)	0.0005

^*a*^ Receiver operating characteristic (ROC) curve analysis was performed for each tRNA to assess diagnostic performance in distinguishing HIV-1 or HIV-2 infection from uninfected controls (*n *= 20 per group). Area under the curve (AUC) values, 95% confidence intervals (CI), and *P*-values are shown. tRNAs with AUC ≥ 0.700 and < 0.05 were considered to have diagnostic potential. Nuclear-encoded tRNAs are shown in regular text; mitochondrial-encoded tRNAs are prefixed with “mt.” The tRNAs included in Model-I (HIV-1 detection) and Model-II (HIV-2 detection) are indicated in bold. AUC interpretation: 0.90–1.00 = excellent; 0.80–0.90 = good; 0.70–0.80 = fair.

### Establishment of the predictive tRNA models for HIV-1 and HIV-2 detection

**Fig 6:**
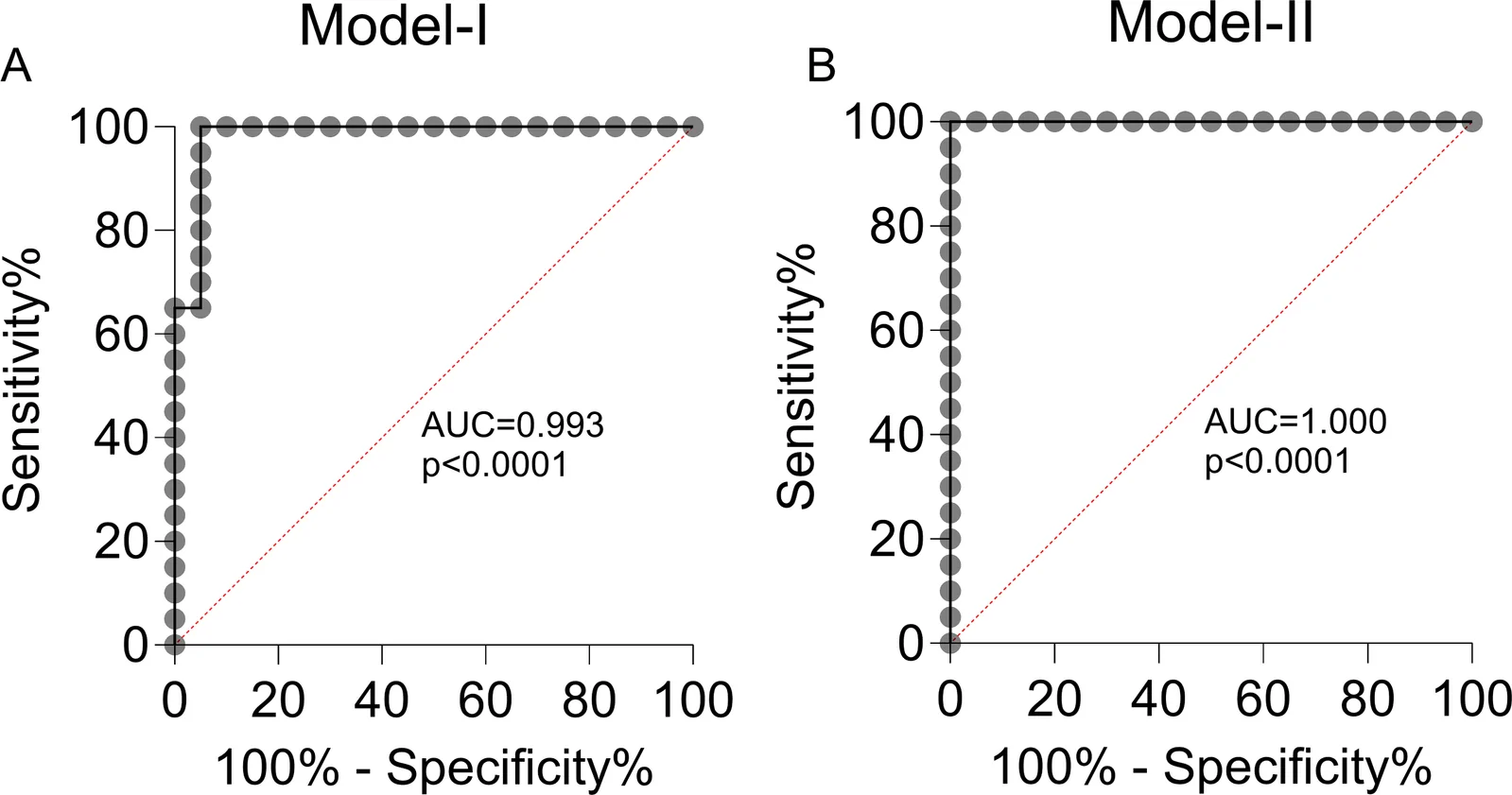
Receiver operating characteristic (ROC) curve analysis of tRNA panels in the independent verification stage. (**A**) Model-I [Logit (HIV-1) = 0.740 + 0.052 × Glu-TTC − 0.106 × Pro-TGG − 0.053 × Val-AAC + 0.101 × mt-Leu-TAA] in HIV-1 vs uninfected controls. (**B**) Model-II [Logit (HIV-2) = − 0.981 − 0.05 × Leu-TAA + 0.052 × mt-Asp-GTC − 0.143 × mt-Ala-TGC + 0.185 × mt-Lys-TTT − 0.126 × mt-LeuTAA + 0.062 × GlyCCC-1] in HIV-2 vs uninfected controls.

A logistic regression analysis was performed to determine the most suitable combination of tRNAs for the diagnosis of HIV-1 or HIV-2 infection. The results of the analysis indicated that a linear combination of the expression levels of Glu-TTC, Pro-TGG, Val-AAC, and mt-Leu-TAA, produced the best model for diagnosing HIV-1 infection (Model-I). A different combination of tRNAs, including Leu-TAA, mt-AspGTC, mt-Ala-TGC, mt-Lys-TTT, mt-Leu-TAA, and Gly-CCC-1, produced another model for diagnosing HIV-2 infection (Model-II) (Table 2). The predicted probability of detecting samples from HIV-1-infected individuals using Model-I was calculated as: logit (HIV-1) = 0.740 + 0.052 × Glu-TTC − 0.106 × Pro-TGG − 0.053 × Val-AAC + 0.101 × mt-Leu-TAA. In this equation, the tRNA symbol was replaced with the ∆Ct value of the tRNA. If the result of logit [HIV-1] was greater than or equal to 0.503, the sample was predicted to be from an HIV-1-infected individual. The four tRNA signature panels increased the AUC value to 0.993 (95% CI: 0.994–1.00, *p* < 0.00001; Fig. 6A) with 100% sensitivity and 95% specificity, which is improved compared with the use of each tRNA alone (Tables 1 and 2). Similarly, those tRNAs that were significantly different between HIV-2 and uninfected control (Fig. 5B and Table 1) were included in the logistic regression analysis. The selected tRNAs were grouped into panels designated as HIV-2 diagnosis (Fig. 6B and Table 2). The cutoff values of the diagnostic performances of these models were determined based on the maximum Youden index (HIV-2 ≥0.483 for HIV-2 versus uninfected control). The model demonstrated 100% sensitivity and 100% specificity for distinguishing HIV-2 infection from uninfected controls (Fig. 6B and Table 2). Therefore, both Model-I and Model-II can be used for the detection of HIV-1- and HIV-2-infected individuals compared to uninfected control individuals.

**TABLE 2:** tRNA panels for diagnosis of HIV-1 and HIV-2 in validation data sets

tRNA panel	Targeted disease vs control	Model	Cutoff value	Sensitivity	95% CI sensitivity	Specificity	95% CI specificity
Model-I	HIV-1	Logit (HIV-1) = 0.740 + 0.052 × Glu-TTC − 0.106 × Pro-TGG − 0.053 × Val-AAC + 0.101 × mt-Leu-TAA	≥ 0.503	100%	83.89% to 100.0%	95%	76.39% to 99.74%
Model-II	HIV-2	Logit (HIV-2) = −0.981 − 0.05 × Leu-TAA + 0.052 × mt-Asp-GTC − 0.143 × mt-Ala-TGC + 0.185 × mt- Lys-TTT − 0.126 × mt-LeuTAA + 0.062 × GlyCCC-1	≥ 0.483	100%	83.89% to 100.0%	100%	83.89% to 100.0%

### Accuracy of the tRNA models

**Fig 7:**
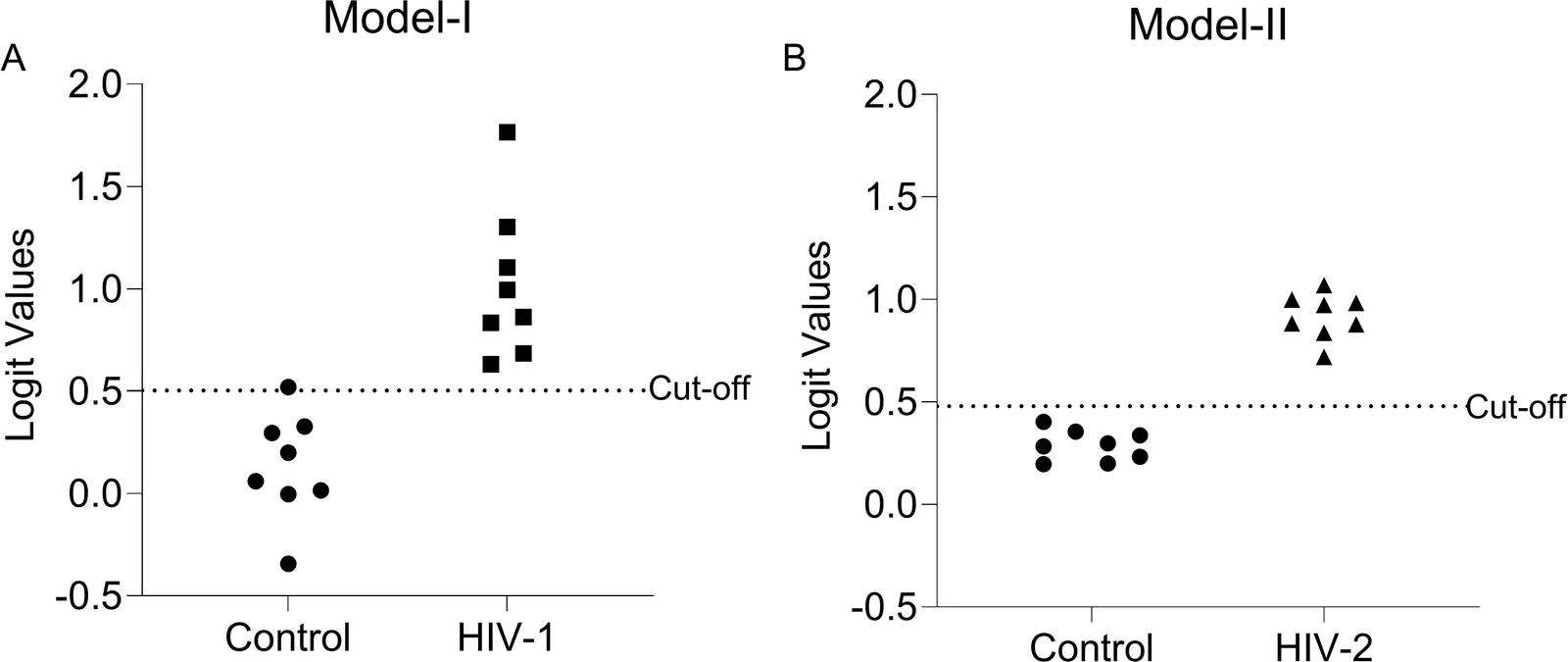
Logistic regression analysis—logit values from the blinded samples. (**A**) The level of plasma tRNAs from eight HIV-1- positive plasma samples and eight uninfected control plasma samples were examined as blinded samples using real-time RT-PCR. Logit values were derived from regression analysis Model-I for detection of HIV-1 infection. The results showed that HIV-1 was detected from uninfected control with 100% sensitivity and 87.5% specificity. (**B**) The level of plasma tRNAs from eight HIV-2-positive plasma samples and eight uninfected control plasma samples were examined as blinded samples using real-time RT-PCR and normalized to 18S rRNA as the internal control. Logit values were derived from regression analysis Model-II for the detection of HIV-2 infection. The results showed that HIV-2 was detected from uninfected normal control with 100% sensitivity and specificity. The normalized ∆Ct values for the individual tRNAs were presented in Fig. S1.

To evaluate the accuracy of diagnostic Model-I and II, we conducted a blinded study to test another 16 independent plasma samples from HIV-1/HIV-2-infected patients (HIV-1 *n* = 8, HIV-2 *n* = 8) and eight uninfected controls. In this study, the patients’ clinical data were not known to us during the evaluation. The sensitivity and specificity were calculated based on the models established in the verification stage. The results showed that Model-I, composed of the four tRNAs (Glu-TTC, Pro-TGG, Val-AAC, and mt-Leu-TAA), correctly discriminated HIV-1 infection from uninfected controls with 93.75% accuracy, 100% sensitivity, and 87.5% specificity (Fig. 7A). In contrast, Model-II [Logit ([HIV-2) = − 0.981 − 0.05 × Leu-TAA + 0.052 × mt-Asp-GTC − 0.143 × mt-Ala-TGC + 0.185 × mt-Lys-TTT − 0.126 × mt-LeuTAA + 0.062 × GlyCCC-1] correctly discriminated HIV-2 infection from uninfected controls with 100% accuracy, sensitivity, and specificity (Fig. 7B).

### Assessment of the specificity of tRNA models

**Fig 8:**
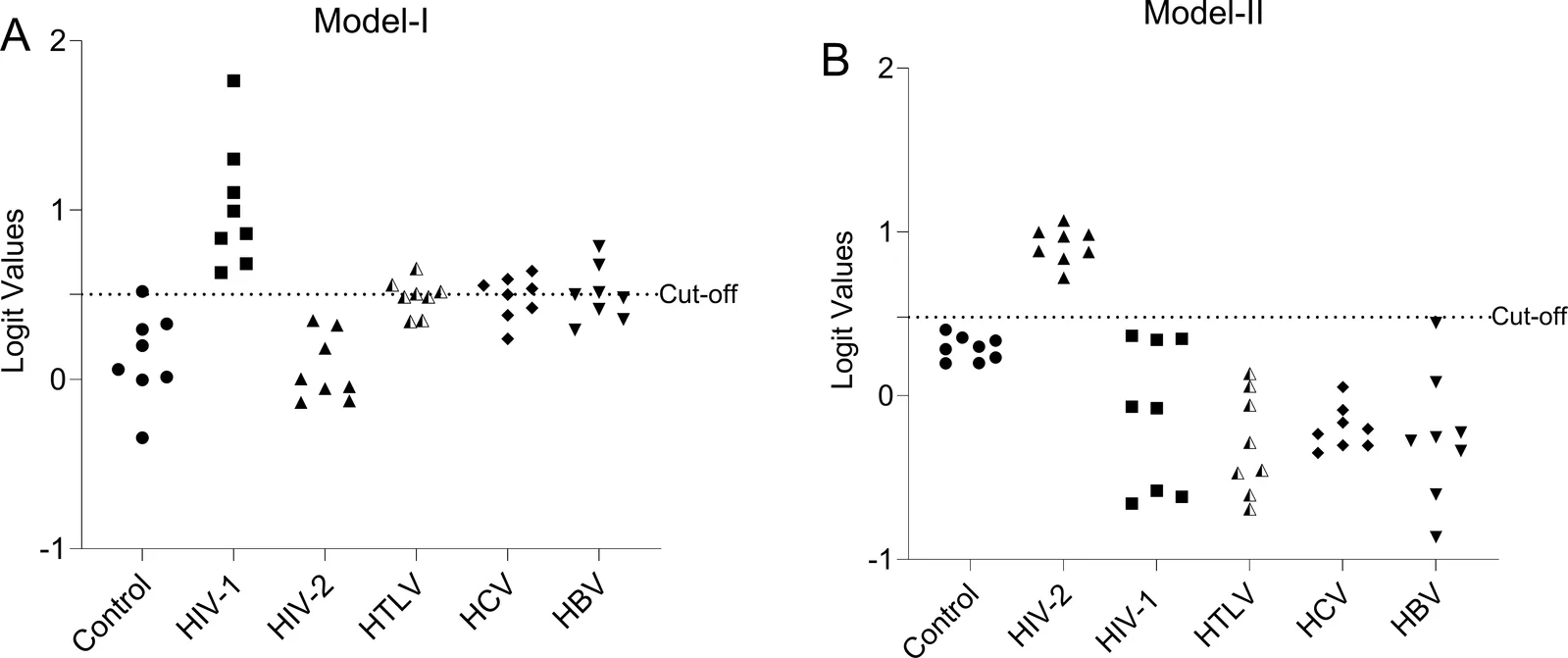
Specificity of the Model-I and Model-II tRNA panels for detection of HIV-1 and HIV-2. Expression of the (**A**) Model-I and (**B**) Model-II tRNA signatures in HIV-1 (*n* = 8), HIV-2 (*n* = 8), HTLV (*n* = 8), HCV (*n* = 8), and HBV (*n* = 8) positive plasma samples and uninfected control donors (*n* =8). The normalized ∆Ct values for the individual tRNAs were presented in Fig. S1.

To determine the specificity of Model-I, plasma samples from HIV-1-infected individuals (*n* = 8), HIV-2-infected individuals (*n* = 8), HTLV-infected individuals (*n* = 8), HBV-infected individuals (*n* = 8), and HCV-infected individuals (*n* = 8) were tested. Results indicated that the Model-I signature of tRNAs was specific to HIV-1 infection, and the plasma samples from HIV-2-infected individuals had signatures similar to those of uninfected control samples (Fig. 8A). However, considerable cross-reactivity was observed with plasma samples from HTLV-, HCV-, and HBV-infected individuals. Four out of eight plasma samples, five out of eight plasma samples, and four out of eight plasma samples from HTLV-, HCV-, and HBV-infected individuals, respectively, were scored as reactive; however, the sensitivity was lower than that observed for samples from HIV-1-infected individuals (sensitivity 50%, 62.5%, and 50% for HTLV, HCV, and HBV, respectively). To assess the specificity of Model-II for HIV-2 infection in a similar way, we tested the expression levels of six tRNAs (Leu-TAA, mt-Asp-GTC, mt-Ala-TGC, mt-Lys-TTT, mt-Leu-TAA, and Gly-CCC-1) in HIV-1- (*n* = 8), HIV-2- (*n* = 8), HTLV- (*n* = 8), HBV- (*n* = 8), and HCV (*n* = 8)-infected individuals and compared them with uninfected control individuals (*n* = 8). The data suggested that the Model-II signature of tRNAs was exclusively associated with HIV-2 infection, as plasma samples from individuals infected with HIV-1, HTLV, HCV, or HBV exhibited signatures similar to those of uninfected control samples (Fig. 8B). The two unrelated viruses, HBV and HCV, had no cross-reactivity with HIV-2, denoting 100% specificity (Fig. 8B). Importantly, there was no cross-reactivity observed for the two related viruses HIV-1 and HTLV, suggesting that Model-II is specific for HIV-2 infection and can distinguish between HIV-2 and other retroviruses.

## DISCUSSION

There are significant biological and clinical differences between HIV-1 and HIV-2. HIV-2 exhibits intrinsic resistance to non-nucleoside reverse transcriptase inhibitors and maintains characteristically lower viral loads than HIV-1, making standard HIV-1- optimized assays insufficient for the accurate detection, quantification, and monitoring of HIV-2 infections. Furthermore, some rapid HIV tests have limitations in differentiating between HIV-1 and HIV-2 infections, which is critical for choosing the appropriate treatment regimen (39). An easily measurable tRNA signature capable of distinguishing HIV-1 and HIV-2 infection in advance of current diagnostic testing may aid clinicians in making the best therapeutic decision.

Our previous work has shown that levels of different non-coding RNAs are altered in plasma during HIV infection (37, 40). Here, we investigated tRNA gene expression signatures to detect HIV-2 infection. Recent research has shown that tRNA can influence cell proliferation, differentiation, apoptosis, and metabolism, in addition to transporting amino acids and participating in the regulation of biological processes of cells (29, 41, 42). Transfer RNA (tRNA) molecules have been recognized as novel non-invasive biomarkers with high sensitivity and specificity, making them promising candidates for improving cancer diagnosis, prognosis, and personalized therapy strategies (43–45). In recent years, the relationship between HIV-1 infection and tRNA gene expression has attracted attention (46). The cellular tRNA pool is involved in the translation of HIV-1 genes, which share host codon use (33, 47). Some studies indicate that HIV-1 may impact the host’s tRNA pool (33). While previous reports have examined the role of tRNAs during HIV-1 infection, none so far have assessed the capacity of circulating tRNA to detect HIV-2 infection as well as to differentiate HIV-2 from HIV-1 infection. Diagnosis of HIV-2 infection is rare in the USA; data from the U.S. National HIV Surveillance System on a subset of new HIV diagnoses showed that <0.1% of HIV infections were attributable to HIV-2 infection from 2010 to 2017 (48). In this study, we have developed a panel of tRNAs (Model-II) that can detect HIV-2 infections with 100% sensitivity and specificity.

Multiple studies have shown that specific tRNAs and tRNA-derived fragments (tRFs) are selectively packaged into extracellular vesicles (EVs) at concentrations exceeding their cellular abundance, suggesting active sorting mechanisms (49, 50). EV encapsulation protects tRNAs from RNase degradation in extracellular fluids, explaining the remarkable stability of circulating tRNAs in plasma and serum (51). Free RNA molecules are rapidly degraded by abundant extracellular RNases, whereas EV-protected RNAs remain stable for days. HIV-infected cells release EVs containing both viral components (*Gag*, *Nef*, *TAR RNA*) and host RNAs, including microRNAs, long non-coding RNAs, and potentially tRNAs (52, 53). These EVs can transfer functional RNAs to recipient cells, influencing immune responses and potentially facilitating viral spread. Given the distinct pathogenic characteristics of HIV-1 and HIV-2 (different replication kinetics, immune activation levels, and cellular tropism), it is plausible that these viruses induce different EV release patterns and cargo compositions, which could explain the observed difference in tRNA profile. Our initial study of 88 tRNA genes by tRNA PCR array identified 18 tRNAs as promising targets for further analysis. In further studies of independent samples, 11/18 tRNAs were confirmed to be differentially expressed in HIV-1 infection, and 17/18 tRNAs were confirmed to be differentially expressed in HIV-2 infection compared with uninfected normal controls. A single biomarker may have limited efficacy for differentiation of HIV-1 and HIV-2 detection. Those tRNAs with an AUC between 0.7 and 1.00 (*P* < 0.05) were included in a multivariate analysis. Combining tRNAs greatly enhanced detection accuracy and improved both the specificity and sensitivity of the diagnosis, similar to previous findings (37, 40). The tRNA-based Model-II that we have designed employs a panel of tRNAs to detect HIV-2 and differentiate between both HIV-1 infection and uninfected control samples.

The distinct tRNA expression profiles between HIV-1 and HIV-2 infections serve as molecular markers of their divergent pathogenesis, cellular tropism, and metabolic reprogramming. HIV-1 drives severe chronic immune activation (54) and rapid CD4+ T cell depletion via mitochondria-mediated apoptosis (55), which corresponds with the downregulation of specific host tRNAs, including mt-Lys-TTT and tRNA Leu-TAA. Conversely, HIV-2 exhibits delayed clinical progression and utilizes the Vpx accessory protein to expand its tropism into myeloid lineages (56, 57), fundamentally altering the cellular baseline and metabolic origin of released tRNAs. Both lentiviruses differentially manipulate host tRNA pools to accommodate virus-specific codon usage biases; for instance, HIV-1 critically depends on tRNA Leu-TAA for the programmed ribosomal frameshifting required for Gag-Pol polyprotein synthesis (58). A defining hallmark of the HIV-2 signature is the distinct upregulation and predominance of mitochondrial tRNAs—specifically, mt-Asp-GTC, mt-Ala-TGC, mt-Lys-TTT, and mt-Leu-TAA. This robust expression of mitochondrial tRNAs in HIV-2, contrasted with their depletion in HIV-1, could reflect the preservation of oxidative phosphorylation, attenuated viral-induced mitochondrial dysfunction, and slower cell death kinetics, underscoring fundamentally different host-pathogen metabolic interactions. In this study, plasma levels of Pro-TGG, Glu-TTC, Val-AAC, and Leu-TAA tRNAs were significantly elevated in both HIV-1 and HIV-2 infections compared to healthy controls. The tRNA Pro-TGG plays a crucial role in the retroviral life cycle, particularly in reverse transcription and virion assembly (59). Additionally, the matrix (MA) domain of Gag protein binds to tRNA Glu-TTC and Val-AAC, which regulate viral assembly (60, 61). During translation, −1 programmed ribosomal frameshifting depends on tRNA Leu-TAA, which reads UUA codons at slippery sites in HIV-1 mRNAs to produce the Gag-Pol polyprotein (58). Notably, mitochondrial-encoded tRNAs, including mt Lys-TTT, mt Leu-TAA, mt-Ala-TGC, and mt-Asp-GTC, were specifically upregulated in HIV-2-infected individuals, while these same tRNAs were either downregulated or showed no significant changes in HIV-1-infected individuals, highlighting differences in the regulation of host gene expression during HIV-1 and HIV-2 infection.

Our studies have focused on identifying host-based tRNAs as biomarkers to differentiate HIV-1 and HIV-2 infection; however, most of the reported work on HIV-1 and HIV-2 diagnosis has focused on viral markers (62–64). We have developed a model equation using the tRNAs in Model-II (Logit (HIV-2) = −0.981 − 0.05 × Leu-TAA + 0.052 × mt-Asp-GTC − 0.143 × mt-Ala-TGC + 0.185*mt-Lys-TTT − 0.126 × mt- Leu-TAA + 0.062 × Gly-CCC-1) and determined a threshold value (≥0.483) for detecting HIV-2 infection. In the model specificity test phase, we successfully identified HIV-2 infection using the tRNA model. We correctly identified eight out of the eight HIV-2-infected samples (100% sensitivity) and eight out of eight uninfected normal control samples (100% specificity) using the tRNA Model-II. Further, we evaluated the specificity of the tRNAs in Model-II by testing plasma from patients infected with HIV-1, HTLV, HCV, and HBV. The results showed that the signature levels of Model-II were significantly lower in these groups compared to the threshold level, and the signature was similar to that of uninfected individuals. Thus, in the future, after additional large-scale validation studies, we may be able to develop a new tRNA panel and set a threshold value to detect HIV-2 infection based on host tRNAs when viral markers may not be detected.

The extensive use of pre-exposure prophylaxis (PrEP) and post-exposure prophylaxis (PEP) for HIV prevention and treatment involves antiretroviral medications that inhibit HIV replication. This inhibition can result in undetectable levels of HIV RNA (viral load) and can delay or suppress seroconversion (the development of detectable HIV antibodies). Conventional HIV tests, which mainly identify HIV antibodies or viral RNA, may produce false-negative outcomes in recently infected individuals using PrEP or PEP (16, 18, 65). Assays based on host biomarkers, which recognize specific cellular responses to HIV infection, might be capable of detecting early infection prior to the virus being identifiable by standard tests or before seroconversion occurs. By identifying these biomarkers, it may be feasible to detect HIV infection at an earlier stage in individuals on PrEP or PEP, allowing for timely intervention and treatment (37, 40).

High-accuracy HIV-2 diagnostics are vital in West Africa (1%–5% prevalence) to manage dual infections (1%–10%) and prevent treatment failure (66). Robust assays fill critical diagnostic gaps in endemic regions (1, 21). Ongoing migration from regions where HIV-2 is endemic maintains the clinical relevance of this strain in the United States and Europe. The implementation of specialized diagnostic algorithms is essential to resolve frequent misidentifications and ensure appropriate clinical management (1, 2, 5). Differentiation is important because HIV-2 is intrinsically resistant to non-nucleoside reverse-transcriptase inhibitors (NNRTIs), a common class of HIV-1 drugs. Treating an HIV-2 patient with these drugs can lead to treatment failure (67–69).

Despite the promising findings of this study, several limitations must be acknowledged. First, this study relied on commercially obtained archived plasma samples, which, while appropriate for proof-of-concept biomarker discovery (70), may not fully capture the clinical heterogeneity of HIV-2 infection. As these samples likely represent chronic infection, tRNA expression profiles during acute or early-stage HIV-2 infection remain to be characterized. Second, the relatively small sample size, although adequate for initial proof-of-concept discovery, limits the statistical power and generalizability of the current findings. Third, incomplete demographic metadata—including age, sex, race/ethnicity, mode of transmission, CD4 count, and geographic origin—limits our ability to control for potential confounding variables. This is particularly relevant given that tRNA expression is known to be altered in various pathological conditions, most notably neoplastic diseases, where tRNA upregulation supports the increased translational demands of rapidly proliferating cells. Although the samples were reportedly obtained from individuals without known malignancies, the possibility of undiagnosed conditions or co-morbidities that may influence circulating tRNA profiles cannot be entirely excluded.

Future studies should address these limitations through larger, multi-center validation cohorts that include prospectively collected samples with comprehensive clinical and demographic annotation. Given that HIV-2 is predominantly endemic to West Africa and differs from HIV-1 in transmission dynamics, disease progression, and affected populations, validation cohorts should prioritize geographic diversity—particularly from West African populations—and include samples matched for age, sex, race/ethnicity, and mode of transmission to rigorously establish the clinical utility of the tRNA biomarker panel. While our study employed relative quantification using the ΔCt method with 18S rRNA normalization, which is appropriate for biomarker discovery, future clinical validation studies should incorporate absolute quantification using synthetic tRNA standards or digital droplet PCR (ddPCR) to establish precise copy numbers per unit volume and enable assay standardization across laboratories. Absolute quantification will allow establishment of clinical cutoff values in absolute terms (e.g., copies/mL plasma), improve inter-laboratory reproducibility, and support the development of reference materials for quality control. In conclusion, the circulating tRNA panel (Model-II) effectively detects HIV-2 infection. Our specificity testing against other bloodborne viral infections (HTLV, HBV, HCV) demonstrated no cross-reactivity with Model-II, suggesting that the tRNA signature is specific to HIV-2 rather than reflecting general viral infection or immune activation. Nevertheless, future studies should systematically evaluate tRNA expression in individuals with various malignancies, autoimmune diseases, and metabolic disorders to fully establish the clinical specificity of our tRNA biomarker panel and define appropriate clinical contexts for its use. In the target identification stage of this study, we only analyzed 88 tRNAs using PCR Arrays. However, over 500 human tRNA genes can be detected through deep sequencing of tRNA transcripts (tRNA-Seq) (71, 72). Future identification of additional key tRNAs via tRNA-Seq could enhance the current models, potentially enabling development of a clinical model for early HIV-1 and HIV-2 detection and disease progression monitoring.

## Data Availability

The data sets supporting the conclusions of this article are included within the article and its supplemental material.

## SUPPLEMENTAL MATERIAL

Figure S1 jcm.00378-26-s0001.docx Expression levels of nine circulatory tRNAs from Model-I and Model-II in the models' accuracy and specificity testing stage.

Tables S2 and S3 jcm.00378-26-s0002.docx Table S2: List of selected tRNAs from the PCR array for further independent verification. Table S3: Independent verification of tRNAs by quantitative real-time PCR.

Table S1 jcm.00378-26-s0003.xlsx Differentially expressed tRNAs between HIV-1/HIV-2 infected individuals and controls.

ASM does not own the copyrights to Supplemental Material that may be linked to, or accessed through, an article. The authors have granted ASM a non-exclusive, world-wide license to publish the Supplemental Material files. Please contact the corresponding author directly for reuse.
