# Supplementary material for: A novel biomarker signature based on circulating transfer RNA (tRNA) can detect and differentiate HIV-2 infection from HIV-1
Source: J Clin Microbiol. 2026 Aug 31;64(10):e00378-26. doi: 10.1128/jcm.00378-26 (PMC13712997; doi:10.1128/jcm.00378-26)
Supplement: Supplementary file 1 [file jcm.00378-26-s0001.docx]

**Supplementary Figure 1:** **Expression levels of nine circulatory tRNAs from model-I and model-II in the models accuracy and specificity testing stage**. The level of plasma tRNAs in HIV-1 infected individuals (n=8), HIV-2 infected individuals (n=8), HTLV infected individuals (n=8) HCV infected individuals (n=8) and HBV infected individuals (n=8) were tested using real-time RT-PCR and normalized with 18s rRNA. Comparison of the tRNA expression profiles in plasma samples from HIV-1, HIV-2, HTLV, HCV and HBV infected individuals with the uninfected healthy individuals (*p < 0·05; **p < 0·01, ***p < 0·001, ****p < 0·0001 and ns=non-significant as calculated using One Way ANOVA test). In the model-I and model-II the individual tRNA symbol was replaced with the ∆Ct value of the tRNA. If the result of logit was greater than or equal to 0.503 for model-I or 0.483 for model-II, the sample was predicted to be from an HIV-1 or HIV-2 infected individual respectively (figure shown in the manuscript as Figure 7 and Figure 8).
