# Supplementary material for: A novel biomarker signature based on circulating transfer RNA (tRNA) can detect and differentiate HIV-2 infection from HIV-1
Source: J Clin Microbiol. 2026 Aug 31;64(10):e00378-26. doi: 10.1128/jcm.00378-26 (PMC13712997; doi:10.1128/jcm.00378-26)
Supplement: Supplementary file 2 [file jcm.00378-26-s0002.docx]

**Table S2 List of selected tRNAs from the PCR-array for further independent verification**

| **No.** | **Name of t-RNA** | **Log_2_(Fold Change) *** | | **p-value **** | |
| --- | --- | --- | --- | --- | --- |
|  |  | **HIV-1** | **HIV-2** | **HIV-1** | **HIV-2** |
| 1 | Asn-ATT | 2.207227389 | 3.024272919 | 0.020570275 | 0.004983839 |
| 2 | Glu-TTC | -0.454106172 | 4.602651 | 0.727025869 | 0.049998921 |
| 3 | Gly-CCC-1 | -4.144041379 | -5.62434 | 0.000501008 | 0.055164594 |
| 4 | Gly-CCC-2 | 2.150698344 | 2.357094 | 0.010171206 | 0.007335081 |
| 5 | Leu-TAA | 2.817157904 | 2.424573421 | 0.000117342 | 0.000500304 |
| 6 | Pro-TGG | -1.306827386 | -0.94191 | 0.091554824 | 0.505424598 |
| 7 | Ser-GGA | 3.5705568 | 3.385474682 | 0.000139664 | 0.000637016 |
| 8 | Ser-TGA | 2.493141333 | 2.083668232 | 0.00019704 | 0.001004286 |
| 9 | Sup-CTA | 3.174781958 | 2.765309 | 0.000085273 | 0.00032719 |
| 10 | Val-AAC | 3.882409255 | 3.29068327 | 0.000304479 | 0.001469268 |
| 11 | mt-Ala-TGC | -8.528977712 | 1.359771729 | 0.000134382 | 0.453720444 |
| 12 | mt-Asp-GTC | -7.055293878 | -0.033887386 | 0.001236841 | 0.981178572 |
| 13 | mt-Cys-GCA | -7.08686765 | 2.924196243 | 0.000616843 | 0.06337213 |
| 14 | mt-Gly-TCC | -6.117378553 | 3.27118969 | 0.000112409 | 0.0367638 |
| 15 | mt-Leu-TAA | -7.308892091 | 2.613584518 | 0.000346268 | 0.02708907 |
| 16 | mt-Leu-TAG | -6.698860486 | 2.904107094 | 0.000164165 | 0.021577873 |
| 17 | mt-Lys-TTT | -8.21691974 | 3.450953484 | 0.000979169 | 0.054352894 |
| 18 | mt-Met-CAT | -7.851846536 | 1.853414059 | 0.000257509 | 0.081525613 |

Some of the differentially expressed tRNAs associated with HIV-1/HIV-2 infection were selected for further independently verified. The selection criteria were defined to meet the following requirements: 1) Ct values <37 either in HIV-1 infected samples or HIV-2 infected samples or controls to ensure stable detection; 2) relative fold change ≥2 (upregulation) or ≤0·5 (downregulation) between the HIV-1 infected samples or HIV-2 infected samples and control groups.

^*^Log_2_ (Fold Change) is calculated by the ΔCt method using average Ct values of RNU6 and SNORD43 as a control

^**^p-value were calculated based on a student’s-test

**Table S3: Independent verification of tRNAs by quantitative real time PCR**

| **No.** | **Name of t-RNA** | **Sample Group^⁋^** | | | **p-value*** | |
| --- | --- | --- | --- | --- | --- | --- |
|  |  | **Control (n=20)** | **HIV-1 (n=20)** | **HIV-2 (n=20)** | **Control vs HIV-1** | **Control vs HIV-2** |
| 1 | Asn-ATT | 12.21±1.69 | 6.80±2.85 | 5.90±1.48 | < 0·0001 | < 0·0001 |
| 2 | Glu-TTC | 12.55±1.44 | 8.69±2.17 | 8.29±2.16 | < 0·0001 | < 0·0001 |
| 3 | Gly-CCC-1 | 4.50±1.57 | 4.62±2.49 | 6.60±0.79 | 0.995 | 0.0012 |
| 4 | Gly-CCC-2 | 9.24±1.19 | 3.56±3.96 | 2.17±1.87 | < 0·0001 | < 0·0001 |
| 5 | Leu-TAA | 14.19±1.61 | 8.85±2.08 | 7.53±2.08 | < 0·0001 | < 0·0001 |
| 6 | Pro-TGG | 12.07±2.92 | 7.12±2.47 | 8.78±3.02 | < 0·0001 | 0.0015 |
| 7 | Ser-GGA | 3.70±3.03 | -0.53±1.70 | -0.93±2.22 | < 0·0001 | < 0·0001 |
| 8 | Ser-TGA | 16.87±2.32 | 10.86±2.96 | 9.90±2.14 | < 0·0001 | < 0·0001 |
| 9 | Sup-CTA | 5.90±2.03 | 5.26±2.61 | 2.24±1.80 | 0.7325 | < 0·0001 |
| 10 | Val-AAC | 8.14±3.82 | 0.80±5.90 | 2.67±5.95 | 0.0002 | 0.0057 |
| 11 | mt-Ala-TGC | 5.51±1.93 | 4.55±2.31 | 2.18±1.51 | 0.3296 | < 0·0001 |
| 12 | mt-Asp-GTC | 5.40±2.42 | 5.76±3.35 | 3.45±2.60 | 0.9689 | 0.0957 |
| 13 | mt-Cys-GCA | 5.75±2.03 | 5.25±2.51 | 2.06±1.97 | 0.8510 | < 0·0001 |
| 14 | mt-Gly-TCC | 5.54±2.19 | 6.57±3.09 | 2.46±1.48 | 0.4275 | 0.0003 |
| 15 | mt-Leu-TAA | 4.24±2.01 | 6.04±2.48 | 1.98±1.48 | 0.0210 | 0.0027 |
| 16 | mt-Leu-TAG | 4.82±2.08 | 4.36±2.55 | 1.44±1.66 | 0.8711 | < 0·0001 |
| 17 | mt-Lys-TTT | 3.22±2.00 | 5.01±2.07 | 1.41±1.51 | 0.0113 | 0.0106 |
| 18 | mt-Met-CAT | 3.27±2.19 | 5.24±1.87 | 0.99±1.43 | 0.0042 | 0.0008 |

^⁋^ Data are n=total number of samples, mean ± SD of normalized Ct values

*p-value were calculated based on one-way ANOVA test
